# Efficacy of mineralocorticoid receptor antagonists in postmyocardial infarction patients with or without left ventricular dysfunction: A meta-analysis of randomized controlled trials

**DOI:** 10.1097/MD.0000000000013690

**Published:** 2018-12-21

**Authors:** Yan Xu, Zhiqiang Qiu, Renqiang Yang, Yanqing Wu, Xiaoshu Cheng

**Affiliations:** a Department of Cardiovascular, Institute of Cardiovascular Disease; b Department of Orthopedics, Second Affiliated Hospital of Nanchang University, Nanchang, Jiangxi, China.

**Keywords:** left ventricular dysfunction, meta-analysis, MRAs, myocardial infarction, randomized clinical trials

## Abstract

**Background:**

There is heated debate about the benefits of using mineralocorticoid receptor antagonists (MRAs) in addition to standard therapy in patients admitted for myocardial infarction (MI) with or without left ventricular dysfunction (LVD).

**Methods:**

Randomized controlled trials (RCTs) were scanned by a formal search of electronic databases (PubMed, EMBASE, Cochrane Library, Ovid, and clinical trials) from their inception to April 2018. A meta-analysis was conducted using Review Manager 5.3 to identify studies reporting the efficacy of MRAs use in post-MI patients with or without LVD.

**Results:**

Thirteen RCTs involving 11,365 individuals were eligible for this study. MRAs treatment reduced all-cause mortality by 16%, cardiovascular death by 16%, and death from heart failure (HF) by 22% in post-MI patients. MRAs use reduced all-cause mortality by 13% and cardiovascular death by 15% in post-MI patients with LVD, but there was no significant difference in all-cause mortality and cardiovascular death in post-MI patients without LVD (relative ratios [RR] 0.83, 95% confidence interval [CI] 0.26–2.69, *P* = .76, I^2^ = 0%; RR 1.01, 95% CI 0.33–3.09, *P* = .99, I^2^ = 0%). In 6 RCTs involving post-MI patients, MRAs treatment had a significant effect on improving left ventricular ejection fraction (LVEF) (mean difference 3.33, 95% CI 0.91–5.75, *P* = .007, I^2^ = 94%). Patients treated with MRAs did not show a decrease in recurrent MI or repeat revascularization compared with patients treated without MRAs (RR 0.95, 95% CI [0.80–1.12], *P* = .54, I^2^ = 0%; RR 1.09, 95% CI [0.79–1.50], *P* = .61, I^2^ = 0%). However, MRAs treatment significantly increased the incidence of hyperkalemia compared with patients treated without MRAs (RR 2.05, 95% CI [1.60, 2.61], *P* < .00001, I^2^ = 49%).

**Conclusion:**

MRAs treatment reduced all-cause mortality, cardiovascular death, and death from HF in post-MI patients. MRAs treatment also demonstrated a significant improvement in LVEF. MRAs reduced cardiovascular death and all-cause mortality in patients with LVD. Eplerenone significantly reduced all-cause mortality and cardiovascular death in post-MI patients. However, MRAs failed to show any cardiovascular benefit in post-MI patients without LVD.

## Abstract 2

Supplemental Digital Content is available in the text

## 1 Introduction

Aldosterone, a mineralocorticoid receptor agonist, is synthesized primarily in the adrenal gland and is part of the renin–angiotensin–aldosterone system. Mineralocorticoid receptor antagonists (MRAs), such as spironolactone, eplerenone, canrenoate, and finerenone, are often used to manage chronic and congestive heart failure (HF).^[1]^ MRAs have been shown to reduce mortality and hospitalization in HF patients with reduced ejection fractions, and they might be beneficial for delaying HF with preserved ejection fraction progression.^[2,3]^

Experimental and clinical data have shown that plasma aldosterone levels were significantly higher in myocardial infarction (MI) animals/patients.^[4,5]^ Extensive evidence indicates that aldosterone's main actions on post-MI hearts include cardiac hypertrophy, fibrosis, and increased inflammation and oxidative stress, all of which exacerbate post-MI HF progression.^[6–9]^ Consequently, a number of trials have highlighted the significant benefits of MRAs therapy in post-MI patients.^[10–11]^

In the EPHESUS trial (Eplerenone Post-Acute Myocardial Infarction Heart Failure Efficacy and Survival Study), eplerenone significantly reduced all-cause mortality, cardiovascular mortality and hospitalization in postacute MI patients with left ventricular dysfunction (LVD).^[10]^ However, spironolactone failed to show cardiac benefits in post-MI patients in the ALBATROSS trial (Aldosterone Lethal effects Blocked in Acute myocardial infarction Treated with or without Reperfusion to improve Outcome and Survival at Six months follow-up), where the incidence of hyperkalemia was significantly higher in the MRAs group than in the non-MRAs group.^[12]^ The REMINDER trial (Role of Eplerenone in acute Myocardial Infarction-Double-blind, Early treatment initiation, Randomized, placebo-controlled, multicenter study), meanwhile, found no significant differences in cardiovascular mortality, HF, and arrhythmia between the eplerenone group and the placebo group in ST-elevation MI (STEMI) patients without LVD.^[13]^ Other randomized controlled trials (RCTs) have shown results similar to those of the EPHESUS, REMINDER, and ALBATROSS trials. However, there has been no meta-analysis of the efficacy of MRAs in post-MI patients with or without LVD.

This study aimed to clarify the efficacy of MRAs in post-MI patients with or without LVD, including the effects of MRAs on all-cause mortality, cardiovascular death, death from HF, changes in left ventricular ejection fraction (LVEF), recurrent MI, and the incidence of hyperkalemia.

## 2 Methods

### 2.1 Data sources, search strategy, and selection criteria

RCTs were scanned by a formal search of electronic databases (PubMed, EMBASE, Cochrane Library, Ovid, and clinical trials) from their inception to April 2018. Search terms included: mineralocorticoid receptor antagonists, MRAs, spironolactone, eplerenone, canrenoate, finerenone, myocardial infarction, MI, left ventricular dysfunction, and randomized controlled trial. These terms were combined with the search algorithm, for example, “Mineralocorticoid receptor antagonists and myocardial infarction.” We looked for RCTs that met all of the following criteria: published articles about RCTs to investigate the efficacy and safety of MRAs in post-MI patients, with groups divided into MRAs and non-MRAs; the drugs of interest were spironolactone, eplerenone, canrenoate, and finerenone; we included trials that evaluated the use of these drugs versus placebo or standard control; studies where populations were all acute MI, ST-elevation MI or post-MI patients; all patients had a follow-up time; studies where the data of sample size, numbers of events, mean difference (MD), and standard deviation were reported in the literature; and hyperkalemia >5.5 mmol/L – 1. All studies that met these requirements were considered eligible for this meta-analysis. LVD as documented by an LVEF of 40% or lower on echocardiography, radionuclide angiography, or angiography of the left ventricle.

### 2.2 Review details and ethics

The literature search, study selection, and data extraction were done independently by 2 reviewers (Yan Xu and Zhiqiang Qiu). Discrepancies were resolved by consensus and overseen by a third investigator (Xiaoshu Cheng). Pertinent information was extracted, including reference data (first author, country, publication year, institution, journal, and intervention), number of patients with or without MRAs, changes in LVEF, and clinical outcomes.

The data we used do not include individual patient data, so ethical approval was not required.

### 2.3 Quality assessment

The Modified Jadad score was used to assess the quality of included studies.^[14,15]^ The Modified Jadad score was calculated by assessing randomization (range, 0–2), concealment of allocation (range, 0–2), double blinding (range, 0–2), and withdrawals and dropouts (range, 0–1). The total score ranges from 0 to 7 and was interpreted according to the following criteria: 0 to 4 indicated a low-quality report and 5 to 7 indicated a high-quality report.

### 2.4 Data synthesis and meta-analysis

The efficacy outcomes were all-cause mortality, cardiovascular death, death from HF, changes in LVEF, the frequency of HF, recurrent MI, and repeat revascularization in follow-up periods. The incidence of hyperkalemia was used as the safety outcome. All statistical analyses were performed using Review Manager (RevMan) version 5.3. The Mantel–Haenszel method for fixed effects and the DerSimonian–Laird method for random effects were used to estimate relative ratios (RR) or MD. Study heterogeneity was assessed by the Cochran Q test and I^2^ statistic, and it was considered significant if *P* < .10 for the Q statistic or I^2^ > 50%. When significant heterogeneity was detected, data from the included studies were combined with the random-effects model; otherwise, the fixed-effects model was utilized. Data were presented as RR or MD with 95% confidence intervals (CIs), with 2-tailed *P* values. Statistical significance was set at a *P* < .05 (2-tailed).

## 3 Results

### 3.1 General characteristics of studies included in the meta-analysis

**Figure 1:**
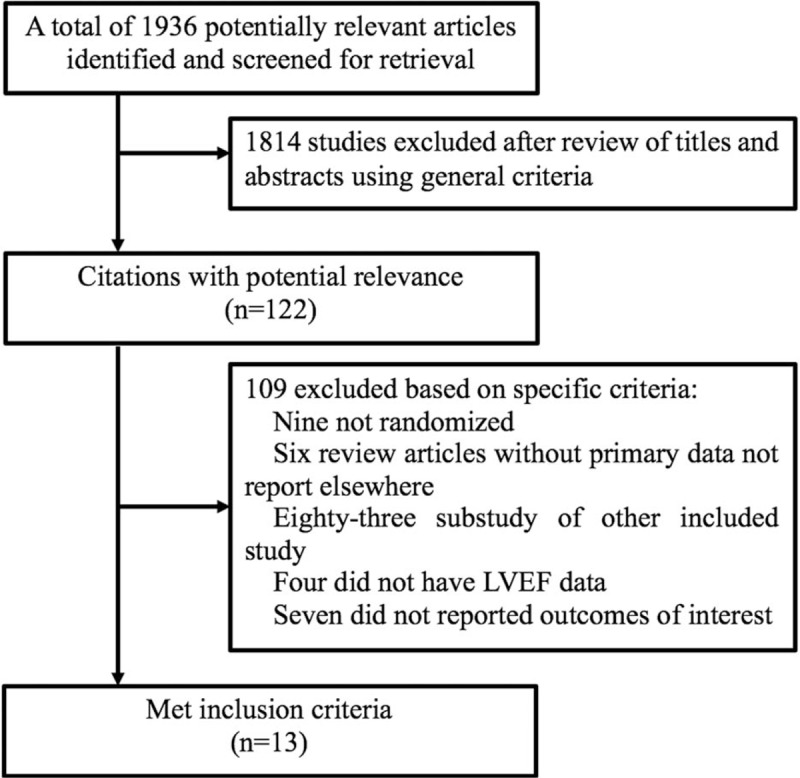
Flow diagram of study identification and selection.

The search strategy found 1936 articles, among which 122 articles met the general inclusion criteria and were reviewed for strict inclusion or exclusion criteria (Fig. 1). Thirteen RCTs were included in the meta-analysis. Eighty-one articles reported the substudy results of the EPHESUS trial in terms of different follow-up periods, clinical outcomes, and etiology. Reports by Pitt 2003 and Iqbal 2014 were included in our meta-analysis.^[10,16]^ Pitt 2003 reported the effects of eplerenone on major clinical outcomes among LVD patients after MI during a 16-month follow-up period. Percutaneous coronary intervention (PCI) was the main therapeutic strategy for MI, this intervention significantly affected the clinical outcomes of MI patients. Iqbal 2014 evaluated the effect of eplerenone administration on HF patients managed with PCI. We extracted the data about repeat revascularization from that article (Iqbal 2014).

The demographics and basic characteristics of patients are described in Table 1. Modified Jadad scores varied by 5 to 7 points, and all of the RCTs included in the meta-analysis indicated a high-quality report. The mean score of the studies was 6.2 points in this meta-analysis.

**Table 1:**
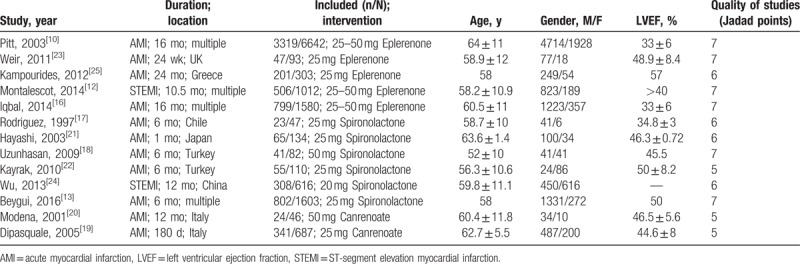
Main characteristics of included studies.

Overall, 12 RCTs involving 11,365 patients were used in the meta-analysis (Iqbal 2014 was the substudy of Pitt 2003, the number of patients was not counted in the meta-analysis). These trials investigated the efficacy and safety outcomes of MRAs use in post-MI patients with or without LVD. Among these trials, patients were treated with spironolactone in 5 trials, eplerenone in 5 trials, canrenoate in 2 trials, and canrenoate plus spironolactone in 1 trial. Eleven RCTs compared all-cause mortality in post-MI patients treated with or without MRAs (n = 11,085). Seven RCTs compared cardiovascular death in post-MI patients treated with or without MRAs (n = 10,487). Four RCTs compared death from HF in post-MI patients treated with or without MRAs (n = 9072). Changes in LVEF were compared in 6 RCTs in post-MI patients treated with or without MRAs (n = 1117). Four RCTs compared recurrent MI (n = 8968) and 3 RCTs compared repeat revascularization (n = 3293) in post-MI patients treated with or without MRAs. Six RCTs compared the incidence of hyperkalemia in post-MI patients treated with or without MRAs (n = 10,265). All RCTs were parallel arm trials and ranged in duration from 1 to 16 months.

### 3.2 Effects of MRAs on all-cause mortality

**Figure 2:**
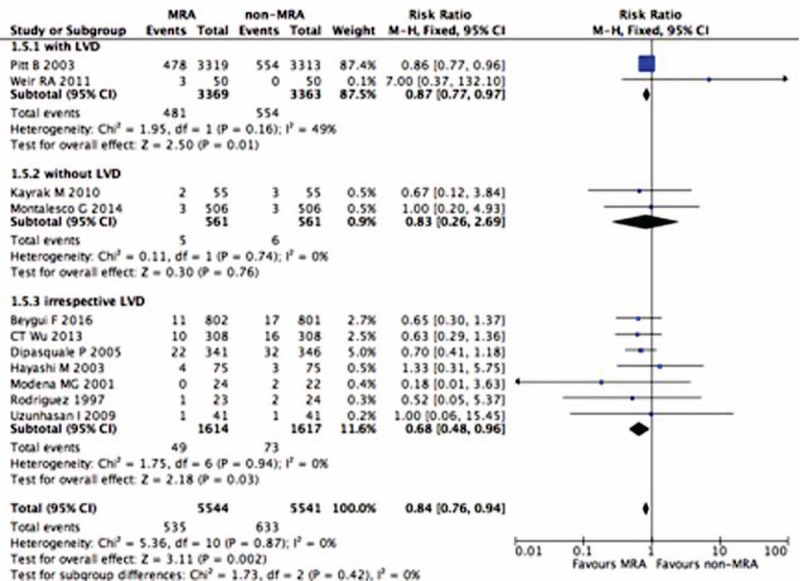
Effect of MRAs on all-cause mortality in post-MI patients with or without LVD. LVD = left ventricular dysfunction, MI = myocardial infarction, MRAs = mineralocorticoid receptor antagonists.

The effects of MRAs on all-cause mortality in post-MI patients were investigated in 11 studies that included 5544 patients treated with MRAs and 5541 patients treated with a placebo or standard controls.^[10,12,13,17–24]^ The all-cause mortality rate in post-MI patients was 9.65% (n = 535/5544) for those treated with MRAs and 11.42% (n = 633/5541) for the placebo or standard control-treated patients. Individuals enrolled in 2 RCTs were post-MI patients with LVD. Individuals enrolled in 2 other RCTs were without LVD. In 7 RCTs, patients were selected irrespective of LVD. Analysis of the overall effects showed that MRAs reduced all-cause mortality by 16% (RR 0.84, 95% CI 0.76–0.94, *P* = .002, I^2^ = 0%) (Fig. 2). A subgroup analysis was conducted for patients with or without LVD. The results showed that all-cause mortality was reduced by 13% in patients with LVD (RR 0.87, 95% CI 0.77–0.97, *P* = .01, I^2^ = 49%) and by 32% in post-MI patients irrespective of LVD (RR 0.68, 95% CI 0.48–0.96, *P* = .03, I^2^ = 0%), but there were no significant differences in all-cause mortality between the MRAs group and the non-MRAs group in post-MI patients without LVD (RR 0.83, 95% CI 0.26–2.69, *P* = .76, I^2^ = 0%). We also did a subgroup analysis according to different MRAs. Patients in 5 trials were treated by spironolactone, in 3 trials by eplerenone, in 2 trials by canrenoate, and in 1 trial by canrenoate plus spironolactone. The results showed that eplerenone significantly reduced all-cause mortality in post-MI patients (*P* = .01, I^2^ = 0%) (Supplemental Fig. 1).

### 3.3 Effects of MRAs on cardiovascular death

**Figure 3:**
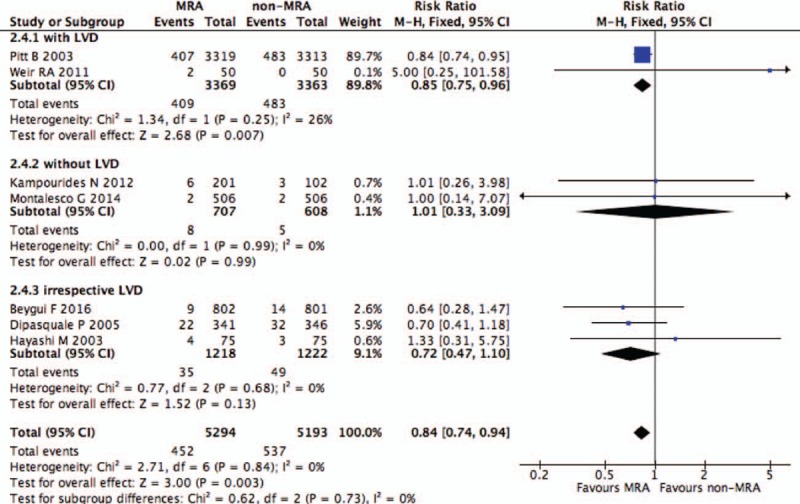
Effect of MRAs on cardiovascular death in post-MI patients with or without LVD. LVD = left ventricular dysfunction, MI = myocardial infarction, MRAs = mineralocorticoid receptor antagonists.

Seven RCTs were evaluated the effects of MRAs on cardiovascular death in post-MI patients (N = 10,487).^[10,12,13,19,21,23,25]^ Cardiovascular death in post-MI patients was 8.54% (n = 452/5294) in those treated with MRAs and 10.34% (n = 537/5193) in those who received a placebo or standard control. Individuals enrolled in 2 RCTs were post-MI patients with LVD, those enrolled in 2 others were without LVD, and those enrolled in 3 others were selected irrespective of LVD. Analysis of the overall effects on cardiovascular death revealed a significant difference between patients who were treated with or without MRAs (RR 0.84, 95% CI 0.74–0.94, *P* = .003) (Fig. 3) without heterogeneous results (I^2^ = 0%). In the overall comparison, the reduction of cardiovascular death with MRAs was greater than in patients who were treated without MRAs. A subgroup analysis was conducted according to patients with or without LVD. The results showed that MRAs use reduced cardiovascular death by 15% in patients with LVD (RR 0.85, 95% CI 0.75–0.96, *P* = .007, I^2^ = 26%); however, there was no significant difference in cardiovascular death between the MRAs group and the non-MRAs group in post-MI patients without LVD or irrespective of LVD (RR 1.01, 95% CI 0.33–3.09, *P* = .99, I^2^ = 0%; RR 0.72, 95% CI 0.47–1.10, *P* = .13, I^2^ = 0%). We also performed a subgroup analysis according to different MRAs. Patients in 1 trial were treated with spironolactone, in 4 trials with eplerenone, in 1 trial with canrenoate, and in 1 trial by canrenoate plus spironolactone. The results showed that eplerenone significantly reduced cardiovascular death in post-MI patients (*P* = .008, I^2^ = 0%) (Supplemental Fig. 2).

### 3.4 Effects of MRAs on death from HF

**Figure 4:**
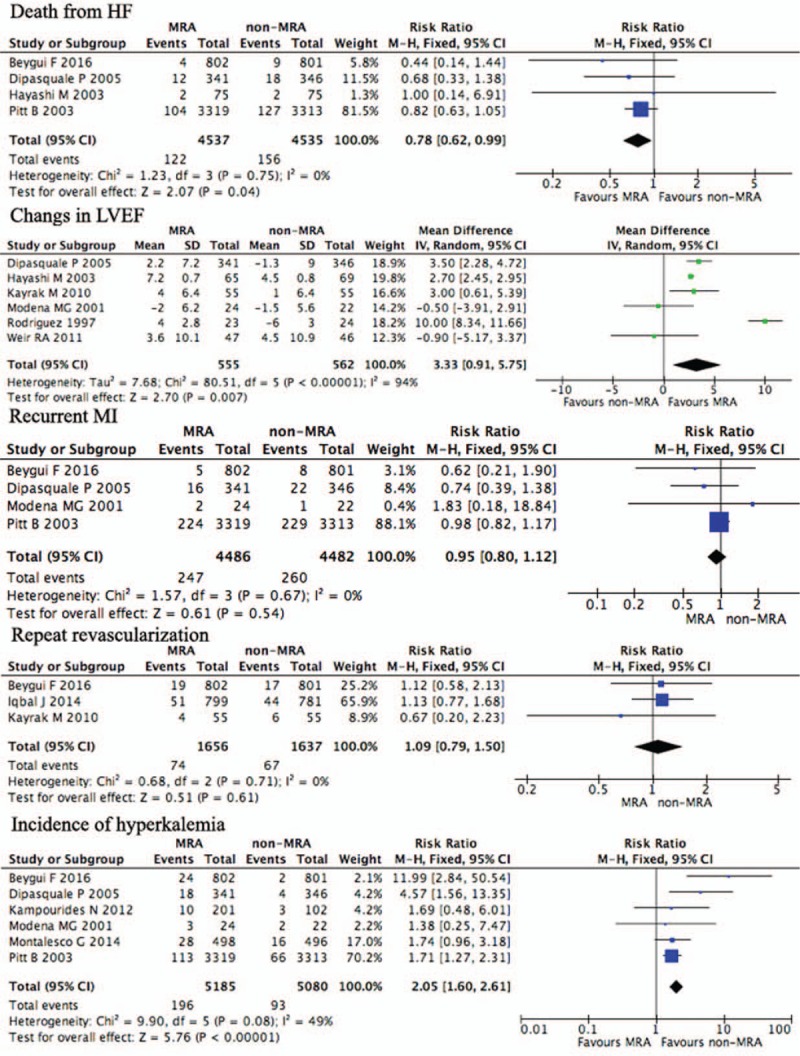
Other outcomes of MRAs use in post-MI patients. MI = myocardial infarction, MRAs = mineralocorticoid receptor antagonists.

Exhaustive data on death from HF were reported by 4 studies (N = 9072).^[10,13,19,21]^ Death from HF was observed in 122 (2.69%) of the 4537 patients belonging to the MRAs group versus 156 (3.44%) of the 4535 patients belonging to the non-MRAs group. The forest plot shown in Fig. 4 summarizes the effects of MRAs on death from HF in post-MI patients. I^2^ for the different studies was 0%, and we used fixed effects to evaluate RR in 2 groups. The rate of death from HF in the MRAs group was significantly lower than that in the non-MRAs group (RR 0.78, 95% CI [0.61, 0.99], *P* = .04) (Table 2).

**Table 2:**
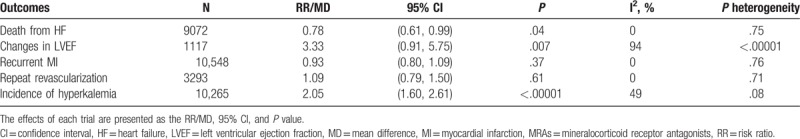
Other outcomes of MRAs use in post-MI patients.

### 3.5 Changes in LVEF in post-MI patients treated with or without MRAs

The effects of MRAs on changes in LVEF in post-MI patients were investigated in 6 studies that included 555 patients treated with MRAs and 562 patients treated without MRAs.^[17,19–23]^ Individuals enrolled in one RCT were post-MI patients with LVD, those enrolled in one other RCT were without LVD, and those enrolled in 4 RCTs were selected irrespective of LVD. The analysis of the overall effects showed a significant difference in changes in LVEF between patients who were treated with or without MRAs (MD 3.33, 95% CI 0.91–5.75, *P* = .007) with high heterogeneous results (I^2^ = 94%). Random effects were used to evaluate RR in 2 groups (Fig. 4, Table 2). Compared to the non-MRAs group, post-MI patients treated with MRAs had improved LVEF.

### 3.6 Effects of MRAs on recurrent MI and repeat revascularization

Four RCTs evaluated the effects of MRAs on recurrent MI (N = 8968),^[10,13,19,21]^ and 3 evaluated repeat revascularization (N = 3293)^[13,16,22]^ in post-MI patients. Recurrent MI in post-MI patients was 5.53% (n = 247/4468) in those treated with MRAs and 5.80% (n = 260/4482) in those who received a placebo or standard control in post-MI patients. The analysis of the overall effects on recurrent MI showed no significant difference between patients who were treated with or without MRAs (RR 0.95, 95% CI 0.80–1.12, *P* = .54) (Fig. 4, Table 2) without heterogeneous results (I^2^ = 0%). Repeat revascularization in post-MI patients was 4.47% (n = 74/1656) in those treated with MRAs and 4.09% (n = 67/1637) in those received a placebo or standard control in post-MI patients. The analysis of the overall effects on repeat revascularization found no significant difference between patients who were treated with or without MRAs (RR 1.09, 95% CI 0.79–1.50, *P* = .61) (Fig. 4, Table 2) without heterogeneous results (I^2^ = 0%).

### 3.7 Incidence of hyperkalemia in post-MI patients treated with or without MRAs

The evaluation of the incidence of hyperkalemia was performed in 6 RCTs, for a total of 10,265 post-MI patients.^[10,12,13,19,20,25]^ The incidence of hyperkalemia was observed in 196 (3.78%) of the 5185 patients belonging to the MRAs group versus 93 (1.83%) of the 5080 patients belonging to the non-MRAs group. The forest plot shown in Fig. 4 summarizes the effects of MRAs on the incidence of hyperkalemia in post-MI patients. I^2^ for the different studies was 49%. There was a significant difference in the incidence of hyperkalemia between post-MI patients treated with or without MRAs (RR 2.05, 95% CI [1.60, 2.61], *P* < .00001) (Fig. 4, Table 2). Overall, compared to those who received a placebo or standard therapy, the rates of hyperkalemia were doubled in patients treated with MRAs.

## 4 Discussion

In our meta-analysis, we evaluated the efficacy and safety of MRAs in post-MI patients from 13 RCTs. MRAs treatment reduced all-cause mortality, cardiovascular death, and death from HF. MRAs treatment also demonstrated a significant improvement in LVEF. There were no significant differences in recurrent MI and repeat revascularization in post-MI patients treated with or without MRAs. MRAs reduced cardiovascular death and all-cause mortality in patients with LVD. However, MRAs failed to show any cardiovascular benefit in post-MI patients without LVD. The incidence of hyperkalemia was increased in the MRAs group.

Many studies in recent decades have established the crucial role of an activated adrenergic system plays in accelerating MI and HF progression.^[4,26]^ Bathgate-Siryk et al^[9]^ demonstrate that β-arrestin-1 played a key role in post-MI HF pathophysiology via actions not only in the heart but also in the adrenal gland, where catecholamines were primarily produced. Indeed, genetic deletion of β-arrestin-1 markedly improved cardiac function, adverse remodeling, aldosterone levels, and cardiac β-adrenergic receptor function during HF progression. The benefits of β-blockers that mitigate or protect the heart from this adrenergic system hyperactivity are also well documented.^[26,27]^ However, the benefits of MRAs used in MI patients require further investigation. Ezekowitz and McAlister^[28]^ found a 20% reduction in all-cause mortality with MRAs use in LVD patients. Bossard et al found that mortality was reduced in the MRAs group versus the non-MRA group in acute MI patients with HF. Among those without HF, the mortality rate was 2.5% in acute MI patients treated with MRAs versus 3.5% among those without MRAs (*P* = .43).^[29]^ Chen et al^[30]^ found that MRAs treatment reduced hospitalization for HF and caused quantifiable improvements in quality of life, diastolic function, and reversal of cardiac remodeling in HF in patients with preserved ejection fractions but did not provide any all-cause mortality benefit. Others studies, in agreement with ours, found that MRAs had all-cause mortality benefits and cardiac benefits in post-MI patients with LVD. Canrenoate is an aldosterone antagonist of the spironolactone group, which is used as a diuretic in Europe. Canrenoate data were extracted from 2 articles, the patients in 2 articles were all from Italy. The all-cause mortality and cardiovascular death results were the same when we excluded the canrenoate data. However, for patients without LVD, large-scale RCTs are needed to further validate the efficacy of MRAs for all-cause mortality.

Experimental animal studies have found that MRAs therapy administered prior to reperfusion reduced MI size.^[31]^ MRAs treatment improved LVEF in post-MI patients in our meta-analysis, this result is similar to those reported by Rodriguez et al,^[17]^ Hayashi et al,^[21]^ and Kayrak et al.^[22]^ We do the sensitivity analysis without Rodriguez's study, I^2^ is 49% when this article was removed, the result also reveal MRAs improve LVEF in post-MI patients (MD 2.56, 95% CI 1.57–3.55, *P* < .00001, I^2^ = 49%). In Ezekowitz's meta-analysis,^[28]^ LVEF significantly improved in HF patients, but no significant LVEF improvement was seen in post-MI patients. The ongoing MINIMISE STEMI trial will investigate whether early MRAs therapy, initiated prior to reperfusion, can reduce MI size and prevent adverse post-MI left ventricular remodeling in STEMI patients treated by PCI.^[32]^

In our meta-analysis, MRAs failed to show benefits for recurrent MI and repeat revascularization in post-MI patients. However, Song et al^[33]^ found that acute MI patients undergoing PCI who received spironolactone had a lower risk of repeat revascularization. Endothelial dysfunction plays an important role in the pathogenesis of coronary artery disease and MI, and Sudano et al^[34]^ conducted an RCT with 42 patients to evaluate the effects of MRAs on vascular health in coronary artery disease patients with preserved ejection fraction. The results showed that MRAs failed to improve endothelial function and other surrogate markers of cardiovascular health.

Vukadinovic et al^[35]^ performed a meta-analysis of 7 trails including 16,065 patients to investigate the true rate of MRAs-related hyperkalemia in placebo-controlled trials. The results showed that hyperkalemia was more frequently observed in MRAs patients (9.3%) than placebo (4.3%). We obtained the same finding that post-MI patients treated with MRAs had an increased the incidence of hyperkalemia compared with those patients treated without MRAs. The rate of hyperkalemia was 5.9% in LVD patients treated with MRAs.^[28]^ Pitt et al^[36]^ found that treatment with spironolactone was associated with an increased the rate of hyperkalemia in HF patients with preserved LVEF (18.7% vs 9.1% in the placebo group). Pitt et al^[37]^ conducted a subanalysis of the EPHESUS trial to evaluate serum potassium and clinical outcomes from eplerenone; their results showed a beneficial effect of eplerenone on all-cause mortality regardless of baseline risk factors for the development of hyperkalemia. In the ATHENA-HF trial, the change in serum potassium was similar between the high-dose spironolactone group (100 mg) and the usual care alone group.^[38]^ It should be emphasized, however, that serum potassium level was closely monitored when using MRAs, especially in elderly patients and those with renal dysfunction.

This study has several potential limitations. First, we were unable to extract unpublished data to analyze outcomes; thus, the results are limited by a paucity of data. More analyses of subgroups separated by age, nationality, and the presence of STEMI or non-STEMI need to be conducted to evaluate the efficacy and safety of MRAs in post-MI patients. Large-scale RCTs are needed to further validate the current results. Second, the follow-ups in the included studies were not all the same, and the period was short. Third, LVEF improvement was not the only marker for heart remodeling; others markers were also used to evaluate heart remodeling, including left ventricular end-diastolic diameter, left ventricular end-diastolic volume index, left atrial volume index, left ventricular end-systolic diameter, and plasma levels of procollagen type III amino-terminal peptide. These data needed to be extracted for analyses to explain the benefits of MRAs for improving cardiac remodeling. Fourth, the patients treated by canrenoate in 2 trials all came from Italy. Last but not least, the number of post-MI patents with LVD was larger than the number of patients without LVD. These are all sources of potential bias.

## 5 Conclusion

In conclusion, based on current evidence, MRAs can reduce all-cause mortality and cardiovascular death in patients with LVD. However, MRAs have failed to show any cardiovascular benefits in post-MI patients without LVD. Large-scale RCTs are needed to further validate the efficacy of MRAs use in post-MI patients without LVD.

## Author contributions

**Conceptualization:** Yan Xu.

**Data curation:** Yan Xu, Zhiqiang Qiu.

**Formal analysis:** Yan Xu, Zhiqiang Qiu.

**Funding acquisition:** Xiaoshu Cheng.

**Investigation:** Renqiang Yang.

**Methodology:** Renqiang Yang.

**Resources:** Renqiang Yang, Xiaoshu Cheng.

**Software:** Zhiqiang Qiu, Yanqing Wu.

**Supervision:** Yanqing Wu, Xiaoshu Cheng.

**Writing – original draft:** Yan Xu, Zhiqiang Qiu.

**Writing – review & editing:** Xiaoshu Cheng.

## Supplementary Material

Supplemental Digital Content
