# Supplementary material for: Efficacy of mineralocorticoid receptor antagonists in postmyocardial infarction patients with or without left ventricular dysfunction: A meta-analysis of randomized controlled trials
Source: Medicine (Baltimore). 2018 Dec 21;97(51):e13690. doi: 10.1097/MD.0000000000013690 (PMC6319977; doi:10.1097/MD.0000000000013690)
Supplement: Supplemental Digital Content [file medi-97-e13690-s001.doc]

Supplemental Figure 1 Effect of different MRAs on all-cause mortality in post-MI patients





Supplemental Figure 2 Effect of different MRAs on cardiovascular death in post-MI patients
